# Hospital-Level COVID-19 Preparedness and Crisis Management in Czechia

**DOI:** 10.3389/ijph.2023.1606398

**Published:** 2023-12-14

**Authors:** Petr Michenka, David Marx

**Affiliations:** ^1^ Third Faculty of Medicine, Charles University, Prague, Czechia; ^2^ Department of Public Health, Third Faculty of Medicine, Charles University, Prague, Czechia

**Keywords:** public health resilience, pandemic preparedness, risk assessment, crisis management, COVID-19

## Abstract

**Objectives:** The COVID-19 pandemic exposed the inadequacy of pandemic preparedness mechanisms worldwide. This study gathered comprehensive data from Czech hospitals, identified possible weaknesses in important areas of crisis preparedness, and quantified changes performed to enhance crisis resilience of healthcare facilities.

**Methods:** Drawing on literature review on pandemic preparedness and hospital crisis management and detailed interviews with hospital representatives, a questionnaire was designed and distributed by email among quality managers of all Czech hospitals. Statistical analysis of their responses was conducted using EZR software. Fisher’s exact test and Kruskal-Wallis test, with *post hoc* testing, were used to assess statistical significance.

**Results:** Achieving response rate of 31.9%, responses from 65 hospitals were analysed. New crisis management policies were necessary in 72.3% of responding hospitals. Furthermore, a majority of the respondents changes indicated the need for changes in policies on general pandemic, human resources and infrastructure and material preparedness.

**Conclusion:** The COVID-19 crisis required significant alterations to previously established hospital crisis management protocols and establishment of new ones. The absence of a unified system for crisis preparedness was noted at hospital and national levels.

## Introduction

Since the first reported case in December 2019, the SARS-CoV-2 virus has presented a significant threat to public health and presented unprecedented challenges to health systems worldwide. It is imperative to thoroughly analyse the response to the COVID-19 pandemic to reduce potential risks and to ensure that corrective measures are implemented across all levels of the public health system. Although initial general analyses [1, 2] of COVID-19 pandemic metrics exist [3], it is crucial to continually collect more detailed data on various aspects of healthcare functioning, including through interviews with those involved and responsible for different levels of crisis response. Only rigorous analyses at all levels of the public health system, and subsequent implementation of necessary correctives and modifications can ensure sufficient preparedness for future pandemics [4] will be sufficient. The study presents findings on pandemic preparedness from the acute inpatient care medical facilities (hospitals) in Czechia, puts them in the context of international literature and identifies areas for further research.

Czechia is a Central European country with approximately 10.7 million inhabitants [5], there are 14 regions with regional cities (serving as a primary administrative centers). The regions are further divided to 76 districts. The Czechia has universal healthcare system and mandatory healthcare insurance with healthcare system highly regulated by the government [6], specifically by the Ministry of Health. The country has 204 hospitals [7], (including acute care hospitals and specialized centers, aftercare hospitals, and psychiatric hospitals). In 2020, there were 6.6 hospital beds and 4.1 medical doctors per 1,000 inhabitants. Tertiary care is provided in university hospitals and regional city hospitals, which usually also provide advanced trauma and advanced ICU care. On the secondary care level, there are district city hospitals with broad spectrum of patients but limited care options. Additionally, there are specialized care centers for various specialties, such as oncology, haematology, and psychiatry, that provide highest level of care in their areas and aftercare hospitals focused on patients with long-term conditions with a need for supportive and rehabilitation care. As of 2019, healthcare expenditure in Czechia was 7.8% of the country’s GDP, amounting to EUR PPP (purchasing power parity) 2,362 EUR *per capita*, which is below the European Union (EU) average of 9.9%—EUR PPP 3,521 *per capita* [6].

During the COVID-19 pandemic, the healthcare system of Czechia was under significant stress. From the beginning of the pandemic in March 2020 (the first confirmed cases of the disease in the country) until July 2023, there were 3,987 SARS-2 caused deaths per 1 million inhabitants (9th highest count in the world, 7th highest count in Europe) and 432,419 cases of SARS-2 infections per 1 million (41st highest count in the world) [8]. The public health functioning in Czechia during the pandemic faced problems encountered worldwide, such as the sudden surge of critical care patients, absolute number of critical care beds and insufficient numbers of personal protective equipment (PPE), but also faced additional country-specific challenges, such as the legislation in force, governmental pandemic decisions and the unsatisfactory state of public health institutions. International COVID analyses [9] could not address all country-specific factors [10]; therefore, additional data were required to analyse the pandemic preparedness and crisis response in Czechia.

Risk assessment can be defined as a “systematic process to comprehend the nature of risk, express and evaluate risk, with the available knowledge” [11]. In the Czech healthcare system, there is no unified risk assessment tool implemented across the whole healthcare sector. The legislation for risk assessment in medical facilities requires the existence of pandemic plans, mass casualty plans, and fire safety plans. However, for all these required plans the government provides only basic content guidance. The lack of systematic control over risk assessment in medical facilities and relative legislative liberty has allowed hospitals to provide only vague guidance on pandemic plan procedures. The sudden onset of the COVID-19 pandemic has challenged the level of preparedness, risk assessment, and crisis management processes in hospitals.

## Methods

From September 2021 to January 2022, principal investigator (PM) conducted eight detailed semi-structured online interviews, which lasted from 30 to 90 min each, with representatives (three quality managers, two head nurses, head of Emergency department, head of Anaesthesiology department, and hospital director) from different hospitals (three University hospitals from different regions, three Regional city hospitals from different regions, two District city hospitals from different districts). The interviews were conducted to identify aspects of COVID-19 pandemic response which were of particular significance in the Czech healthcare environment. Four areas were identified both problematic and prevalent in all interviews: legislation or hospital internal policies, human resources management, infrastructural and supply management issues, and other, uncategorized topics. In order to further supplement information on the gathered topics and complement the scope of the analysis, a semi-systematic literature review was conducted in April 2022 (Supplementary File S1). Drawing on the information gathered from the interviews and literature review, a pilot questionnaire consisting of 52 questions was created (Supplementary File S2) and set up on a commercial survey platform [12]. The questionnaire was divided into five sections based on the identified problematic areas: Demography; General pandemic preparedness; Human resources; Infrastructure and material preparedness; and Other impacts of the pandemic. Eleven open-ended optional questions were included to provide context data in the form of from the survey participants. The questionnaire was first distributed to three pilot hospitals (one University hospital, one Regional city and District city hospital) and revised accordingly. Subsequently, in August and September 2022 (two distribution rounds) the questionnaire with a cover letter explaining the purpose of the survey was distributed through a publicly available email addresses to hospital quality managers. In cases where such addresses were not accessible, the survey was addressed to hospital directors, or to the General Enquiries mailbox. Only medical facilities providing acute inpatient care were surveyed. All other types of facilities, including outpatient facilities, facilities providing scheduled-only inpatient care, and long-term care facilities, were excluded from the study due to their differing systems of functioning, variable facility sizes and limited patient populations which were not relevant for the pandemic response. This method of selection allowed to involve all medical facilities providing acute inpatient care.

Upon survey completion, statistical analyses were conducted in EZR software for medical statistics [13]. Fisher’s exact test was used to assess statistical significance between categorical variables, and Kruskal-Wallis test with *post hoc* testing (Dunn’s test) was used for the risk assessment calculations. All tests were performed at a significance level of 0.05.

### Limitations

Main methodological limitations were both sparse scientific literature available during creation of the questionnaire and low number of primary interviews conducted. Other limitations of the survey are average response rate with high dropout rate which are decreasing the value of the obtained quantitative data. Additionally, the sample size of the open-ended responses was highly diverse, rendering it statistically insignificant for systemic analysis (some of the open-ended responses were reflected in the discussion of the article). It is also essential to emphasize the need for further research specifically focused on the areas covered in the questionnaire to obtain more substantial quantitative data. Another limiting factor is the inability to compare the spectrum of responding hospitals to the bed capacity in Czechia. This limitation arises due to the absence of necessary information in the National Institute of Medical Information registry (this absence was confirmed by the Institute after a demand for the data was made by the authors).

## Results

**FIGURE 1:**
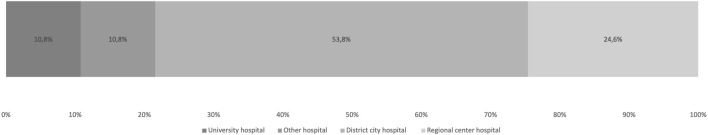
Types of responding hospitals (*n* = 65) (Prague, Czechia, 2023).

Out of the 204 hospitals approached with the questionnaire, 65 hospitals completed to the survey, resulting in a response rate of 31.9% (incomplete responses were excluded, *n* = 61). The highest proportion of respondents was from “District city hospitals” (53.8%, *n* = 35) followed by “Regional city hospitals” (24.6%, *n* = 16) (Figure 1).

### General Pandemic Preparedness

Prior to the onset of the pandemic, 80% of the hospitals (*n* = 52) used an internally developed policy on infectious disease outbreak. Two types of policies were differentiated: policies that involved procedures specified for flu epidemics only (*n* = 9) and policies that involved procedures specified for flu epidemics and for other highly contagious diseases (*n* = 32). Extended infectious disease outbreak policies were more likely to be used *en-bloc* (all parts of the policies used) (66.7% vs. 90.6%; *p* = 0.034). Regardless of the content of the policy, during the pandemic, some changes were required in 86% of cases (*n* = 49). Additionally, in response to the onset of the pandemic, new crisis management policies were created in 72.3% (*n* = 47) of the hospitals. Before the onset of the pandemic, risk assessment was repeatedly and regularly performed in only 10.7% of the hospitals (*n* = 7) and was performed once in 27.7% of the hospitals (*n* = 18). The periodicity of the risk assessment or number of the assessments was not studied. The median number of areas analysed in the risk assessment was statistically significantly higher (*p* = 0.002) in hospitals with regularly repeated risk assessments (11; *n* = 7) than in hospitals with irregularly repeated risk assessments (2.5; *n* = 6). Infectious diseases were covered in 77.8% (*n* = 14) of the assessments.

### Human Resources

System of rotations for employees, defined as “transfer of employees to different units of the same department or to different departments with an aim to enhance the skills and knowledge of individual healthcare worker” for medical doctors was used in 86.2% (*n* = 56) of the hospitals and for non-medical staff in 89.2% (*n* = 58) of the hospitals. Hospitals with an established system of rotations for medical doctors (33.9%, *n* = 22) or non-medical staff (36.9%, *n* = 24) from before the pandemic, were more likely to rotate their staff to other departments and units than the hospitals without such established system (Table 1). The induction of rotated employees into new area of work was mainly “Entirely informal” or “Informal with assigned supervisor” (Table 2). Medical students were involved in healthcare provision in higher extent [14] than before the pandemic in 85.7% of university hospitals, 81.2% of regional city hospitals, 54.3% of district city hospitals and 28.6% of specialized centres (*p* = 0.025). Students of other healthcare professions (nurses, physiotherapists, nutritionists…) were involved in healthcare provision more than before the pandemic in 60% of hospitals (*n* = 39). For students, the analysis of differences by “Type of hospital” variable were not statistically significant. The induction process for students involved in healthcare provision was mainly “Informal with assigned supervisor,” with 61.1% (*n* = 54) for medical students and 61.8% (*n* = 55) for students of other healthcare professions. The alterations to employee care area (work-life balance system, health and wellness benefits, time-off and leaves) were reported in 43.1% of the hospitals (*n* = 28). In 67.9% of the cases (*n* = 19), these alterations will also include changes in psychological support services for its employees. The changes in human resources management were reported in 41.5% of the hospitals (*n* = 27).

**TABLE 1:** Comparison of employee rotations in hospitals with established rotations system from before the pandemic and hospitals without such a system (%) (Prague, Czechia, 2023).

	Medical doctors			Non-medical staff		
Established system of rotations before the pandemic	Only within home department	To other departments	No	Only within home department	To other departments	No
Yes	13.6	86.4	0	4.2	95.8	0
No	18.6	60.5	20.9	9.8	73.2	17.1
*p*-value	0.035			0.040		

**TABLE 2:** Type of induction process for rotated employees; Medical doctors (%; *n* = 49) and Non-medical staff (%; *n* = 51; “Don’t know/Not sure” respondents not included) (Prague, Czechia, 2023).

Medical doctors		Non-medical staff	
Informal with assigned supervisor	Entirely informal	Informal with assigned supervisor	Entirely informal
55.6	35.2	61.8	30.9

### Infrastructure and Material Preparedness

Infrastructure modifications in hospital departments (construction of isolation rooms, installation of specialized equipment - HEPA filters, airlock doors, etc.) were necessary in 69.2% (*n* = 45) of the hospitals, while changes on the hospital spatial arrangement (modification of entrances and changes in other common areas, etc.) in 63.1% (*n* = 41) of the hospitals. In 16.3% (*n* = 8) of the hospitals, all changes made will be preserved after the pandemic is over, and in 77.6% (*n* = 38) at least some changes will be preserved. Hospitals which reported future changes in their development plans (*n* = 22) were more likely to consider construction of multi-purpose buildings, changes in patient admissions processes, and changes in bed management systems, than the hospitals with no such changes planned (*n* = 29) (Table 3). Approximately 90% (*n* = 59) of the hospitals had a real-time bed occupancy monitoring system, but only 64.6% (*n* = 42) reported having an established protocol for patient transfer or referral to other hospitals in cases of insufficient bed capacity. Communication of the stockpile status of medical supplies and medical equipment was primarily limited to hospital employees (Table 4). Only 16.9% (*n* = 11) of hospitals had established protocols for sharing and exchanging medical supplies with other hospitals, and 26.2% (*n* = 17) had protocols for exchanging medical equipment with other hospitals. Nevertheless, 72.3% (*n* = 47) of the hospitals had established protocols for securing medical supplies and equipment during crisis situations. Approximately 60% (*n* = 39) of the hospitals planned changes in their medical supplies’ storage management, while 30.8% (*n* = 20) planned changes in their medical equipment storage management.

**TABLE 3:** Comparison of the hospitals that reported changes in their development plans and included multi-purpose buildings in their development plans, changes in patient admission processes and changes in bed management system with other hospitals (%; *n* = 65) (Prague, Czechia, 2023).

	Multi-purpose buildings			Patient admissions processes			Bed management systems		
Changes in development plans	Yes	No	Don’t know/not sure	Yes	No	Don’t know/not sure	Yes	No	Don’t know/not sure
Yes	54.5	27.3	18.2	59.1	36.4	4.5	68.2	31.8	0
No	27.6	65.5	6.9	37.9	62.1	0	27.6	72.4	0
Don’t know/Not sure	21.4	21.4	57.1	28.6	50	21.4	14.3	35.7	50
*p*-value	0.001			0.035			<0.001		

**TABLE 4:** Communication of medical stockpile status during the pandemic: hospital outreach to employees, other hospitals, and public institutions (%; *n* = 65; “Not sure/do not know” respondents not included) (Prague, Czechia, 2023).

	Medical supplies		Medical equipment	
	Yes	No	Yes	No
Employees	78.46	21.54	66.15	33.85
Other hospitals	46.15	53.85	52.31	47.69
Public institutions	63.08	36.92	61.54	38.46

In addition to the previously discussed areas of crisis preparedness, COVID-19 pandemic appeared as a driving force for change in many other domains of healthcare functioning. The final section of the questionnaire covered these uncategorized areas of the pandemic, identified both during the interviews and in the international literature reviews. While not identified as suitable for deeper analysis conducted in this article, these areas can potentially offer valuable insights into the broader impacts of the pandemic (Table 5 and Supplementary File S3).

**TABLE 5:** Plans for changes based on the experience acquired during the COVID-19 pandemic (%; *n* = 65, “Don’t know/Not sure” respondents not included) (Prague, Czechia, 2023).

Reported area of changes	Yes	No
Infection prevention and control system	71.2	28.8
Management or structure of medical records	48.3	51.7
Placement of medical equipment in the wards	42.4	57.6
Staff training	53.7	46.3
Internal communication strategy	53.6	46.4
Digitalisation of processes or use of telemedicine applications	62.3	37.7
Operational data collection and reporting	70.2	29.8
Communication strategy towards other healthcare providers or Emergency Medical Services providers	47.2	52.8
Public communication strategy	50	50
Co-operation with scientific or private institutions	26.5	73.5

## Discussion

The European Union (EU) mandates member states to evaluate their risk management capabilities every 3 years [15]. Moreover, in response to the pandemic, a policy on resilience of critical infrastructure and critical entity risk assessment has been redefined, with an assessment cycle set to 4 years [16]. However, the definition of critical infrastructure varies across member countries. In Czechia, only hospitals with more than 2,500 acute care beds are classified as critical infrastructure [17]. Therefore, not all hospitals are considered “critical infrastructure,” and the timing of their risk assessment is not standardized across Europe. Typically, these assessments comply with either national legislation requirements [18] or quality assurance agency standards, such as the 2 years timeframe specified by the Joint Accreditation Committee in Czechia [19]. Harmonizing criteria for critical infrastructure, imposing a more rigorous risk assessment requirements, such as the annual review of hospitals’ risk assessments mandated by The Joint Commission [20], or standardizing the risk analysis processes for hospitals across Europe, may yield potential benefits in terms of crisis preparedness.

Notably, in some countries, crisis preparedness protocols beyond mass casualty drills, fire drills and technical preparedness testing (e.g., emergency power supply tests) are neither regularly tested in the field, nor simulated. This may have contributed to the study’s findings that most surveyed Czech hospitals needed to modify their internal policies with focus on pandemic preparedness. After the COVID-19 pandemic experience, challenging infection and prevention control scenarios, such as large-scale pandemics of high transmissibility and/or high-mortality, should also be included in the list of regularly tested situations in all countries. Examples of good practice exist [21–23] and can serve as valuable references. Comparison of the results with available alternatives of risk assessment in medical facilities from other countries [24] suggests a significant need for improvement in the risk analysis area of Czech hospitals, in terms of frequency, form, extent, and detail.

The COVID-19 pandemic has highlighted the importance of adequate staffing and appropriate training of healthcare employees for crisis situations, particularly for those situations that are prolonged in nature [25]. In Czechia, hospitals facing staff shortages adopted strategy of rotating staff from less critical departments to those that were critically understaffed, as recommended by the US Center for Disease Control and Prevention (CDC) [26]. Additionally, some hospitals involved medical and nursing students in healthcare provision during the pandemic [14]. These measures were more prominent in hospitals with a broader spectrum of provided care, such as university and regional city hospitals. However, concerning issues were reported regarding the induction processes for rotated employees (Table 2). From the findings it was evident that some hospitals were not adequately prepared for the implications of staff rotations, and not enough emphasis was placed on the induction processes (Table 2). Despite these challenges, rotating employees proved to be an efficient tool for increasing employee versatility, as well as their job satisfaction and motivation [27], even in non-crisis situations [28, 29]. However, a systematic approach with adequate training and adaptation is critical to ensure safe healthcare processes [30] and continuity of care, and should not be abandoned, especially in critical situations. In the future, efforts can be made to create reserve staff [31] that can serve as a buffer to prevent understaffing, which presents significant challenges even under normal circumstances [32]. The COVID-19 pandemic has also challenged established human resources management routines in the labour market overall [33], while the negative effects of the pandemic, including exhaustion, stress, depression, and burnout, have been more pronounced among healthcare workers [34, 35]. Therefore, it is essential to prioritize and invest into sufficient support networks and psychological care for healthcare employees, to mitigate adverse mental effects and maintain high levels of employees’ wellbeing [36].

The findings of the study revealed that the pandemic has acted as a significant catalyst for infrastructure changes, some of which are likely to become permanent. These changes in development plans will play a strategic role in enhancing future pandemic preparedness and overall resilience of healthcare systems [37]. However, because of the diversity of healthcare systems and the lack of detailed empirical research, it is difficult to establish universally applicable guidelines for infrastructural pandemic preparedness [38]. Consequently, it is advisable to rely on best practice examples and consequently follow general recommendations [39, 40].

The disruption of international supply chains with sudden lack of medical supplies and equipment vital for medical functioning has brought attention to the topic of the management of stockpiles in medical facilities [41]. Previous recommendations on stockpile management from pre-pandemic times [42] will require re-evaluation, as the COVID-19 pandemic highlighted issues with long-lasting global crisis, which is not captured by localized short-term crisis scenarios [43]. The allocation of the stockpile burden between healthcare facilities and nations is a question for international discussion, particularly in situations when national strategies fail [44] and inter-hospital/regional exchange strategies are not established [45]. During the COVID-19 pandemic, international stockpile initiatives proved to be effective and served as a rescue for national failures [46]. Thus, it is a matter of concern whether the current time-limited existence of mechanisms for international cooperation should not be reconsidered, with emphasis being placed on effective interlinking and coordination of stockpile management at all levels of planning [47].

Effective operational data collection and reporting [48], along with a robust IT infrastructure, have proven to be extremely important during the pandemic [49]. The digitalization of healthcare processes [50] and the widespread adoption of telemedicine [51] are likely to play an essential role not only in future pandemics, but also in regular healthcare functioning. Communication strategies towards the public and between healthcare facilities will continue to pose challenges (Table 3). Based on the analyses of time-sensitive data from the pandemic, surge situation protocols for transferring critically ill patients should be redesigned [52] especially with consideration to ethical aspects of the care for patients. There are undoubtedly many other examples of how the thorough review of COVID-19 pandemic response can enhance and improve crisis preparedness efforts. Usefulness of these steps is not limited only to infectious disease outbreaks, but also to other situations challenging public health such natural disasters [53], terrorist attacks [54], and other scenarios with high potential for mass casualties [55]. Maintaining a dedicated effort towards collecting and analysing data on the past and present COVID-19 situation is therefore imperative. The attention to minute details of various aspects of the response has a potential to yield breakthroughs in crisis preparedness, stimulate innovation, and foster the development of a more resilient healthcare system.

### Conclusion

Hospitals in Czechia demonstrated an ability to adapt to the newly emerged crisis, but significant alterations to the established crisis management protocols as well as development of new ones was necessary. The analysis of insights derived from the COVID-19 pandemic has demonstrated that areas of general pandemic preparedness, human resources and infrastructural or material preparedness all posed significant challenges for functioning of the healthcare facilities during the pandemic. The absence of a unified system for crisis preparedness and risk assessment at both hospital and national levels is concerning. Further research is required to analyse remaining aspects of crisis preparedness not primarily covered in this study.
