# Supplementary material for: Hospital-Level COVID-19 Preparedness and Crisis Management in Czechia
Source: Int J Public Health. 2023 Dec 14;68:1606398. doi: 10.3389/ijph.2023.1606398 (PMC10752954; doi:10.3389/ijph.2023.1606398)

**Supplementary file 2**

***Supplementary file 2:*** *The Questionnaire translated from Czech to English*

**Crisis preparedness during COVID-19**

# Page 1 - Introduction

Dear Colleagues,

We kindly request you to participate in a survey that aims to gather information on the crisis preparedness of healthcare providers and facilities during the COVID-19 pandemic. The questionnaire's purpose is to identify problematic areas in both clinical and non-clinical operations, summarize procedures that resolved the situations, aggregate suggestions and findings from practice, and further elaborate the topic in a doctoral dissertation. Completing the entire questionnaire should take about 25 minutes. Your participation is crucial to help us gain insights into the challenges faced by healthcare providers and facilities during the pandemic. We appreciate your time and willingness to help us with this research.

Thank you

XXXX

XXXX

--

*As the entire questionnaire is anonymous, we regret to inform you that the information you enter cannot be saved for intermittent completion. If you prefer to preview the full set of questions before responding, kindly reach out to the principal investigator or click-through the questionnaire and complete it later. We appreciate your cooperation and thank you for Your involement.*


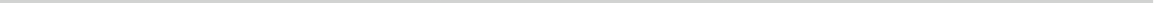


# Page 2 - Demographics

**Type of healthcare provider:**

( ) University hospital

( ) Regional hospital

( ) District hospital

( ) Specialised center

( ) Other provider

**(Optional) Location of the healthcare provider:**

( ) I do not wish to state

( ) Prague

( ) Central Bohemia Region

( ) South Bohemia Region

( ) Pilsen Region

( ) Karlovy Vary Region

( ) Ústí nad Labem Region

( ) Liberec Region

( ) Hradec Králové Region

( ) Pardubice Region

( ) Vysočina Region

( ) South Moravian Region

( ) Olomouc Region

( ) Zlín Region

( ) Moravian-Silesian Region


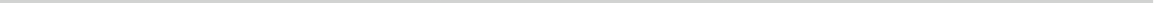


# Page 3 - General Pandemic Preparedness

*Risk analysis/Hazard vulnerability analysis (HVA) is the systematic examination of systems for security weaknesses that pose an acute or potential risk to them, and the examination of the effectiveness of the security measures in use in those systems*

**What type of internal policy on epidemic situation was in use by the healthcare provider in the period before March 2020:**

( ) A generic policy was used (e.g. adapted from abroad or from another institution).

( ) The health care provider used internally developed policy

( ) No policy was used

( ) Don't know, not sure

**This policy specified procedure(s) for:**

( ) Flu epidemics only

( ) Influenza epidemics and other highly contagious diseases

( ) General procedures (without disease specification)

( ) I don't know, I'm not sure

**Has this internal policy been used to organise the operations during the initial phase of the COVID-19 pandemic (March 2020)?**

( ) Yes, completely

( ) Yes, partially

( ) No, not at all.

( ) I don't know, I'm not sure

**Have there been any changes to this policy as a result of the COVID-19 pandemic?**

( ) Yes

( ) No

( ) I don't know, I'm not sure

**(Optional) Please indicate what changes have been made in the internal policy on epidemic situation:**

..........................................................................................................................

..........................................................................................................................

**In response to the onset of the pandemic, did healthcare providers develop new crisis management policies?**

( ) Yes

( ) No

( ) I don't know, I'm not sure

**(Optional) Please indicate what new crisis management policies were developed:**

..........................................................................................................................

..........................................................................................................................

**Has the healthcare provider conducted a hazard vulnerability analysis (HVA)?**

( ) Yes, regularly

( ) Yes, irregularly

( ) Yes, one-off

( ) No

( ) I don't know, I'm not sure

**How many areas were included in the hazard vulnerability analysis?**

...................

**Has the area of infectious diseases been covered in the hazard vulnerability analysis?**

( ) Yes

( ) No

**Has the healthcare provider established a system or protocol for communicating emergency measures to the public?**

( ) Yes

( ) No

( ) I don't know, I'm not sure


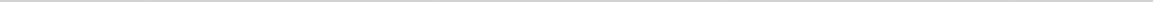


# Page 4 - Human Resources

**Based on the experience gained during the COVID-19 pandemic, is the healthcare provider planning to implement changes in human resource management?**

( ) Yes

( ) No

( ) I don't know, I'm not sure

**(Optional) Please describe what changes in human resources management are planned:**

..........................................................................................................................

..........................................................................................................................

**Did the healthcare provider have a formal rotation system in place for selected healthcare workers prior to the COVID-19 pandemic? (e.g. rotations across different sites to enhance the skills and knowledge of individual healthcare workers)**

|  | Yes | No |
| --- | --- | --- |
| Physicians | ( ) | ( ) |
| Non-physican staff | ( ) | ( ) |

**During the COVID-19 pandemic, was it necessary to implement rotations of healthcare workers?**

|  | Yes, to both other departments and within units of the same department | Yes, only within units of the same department | No |
| --- | --- | --- | --- |
| Physicians | ( ) | ( ) | ( ) |
| Non-physician staff | ( ) | ( ) | ( ) |

**How was the orientation process performed for rotated employees?**

|  | Informally | Informally, with an assigned supervisor | Formally, according to a developed training plan | Not applicable |
| --- | --- | --- | --- | --- |
| Physicians | ( ) | ( ) | ( ) | ( ) |
| Non-physician staff | ( ) | ( ) | ( ) | ( ) |

**During the COVID-19 pandemic, were students involved in clinical operations by the healthcare provider?**

|  | Yes, to a greater extent than  before the pandemic | Yes, to the same extent as  before the pandemic | No |
| --- | --- | --- | --- |
| Students of General Medicine | ( ) | ( ) | ( ) |
| Students of non-physician fields of study | ( ) | ( ) | ( ) |

**How was the orientation process performed for the students involved in clinical operations?**

|  | Informally | Informally with an assigned supervisior | Formally, according to the developed training plan | Not applicable |
| --- | --- | --- | --- | --- |
| Students of  General  Medicine | ( ) | ( ) | ( ) | ( ) |
| Students of non-physician fields of study | ( ) | ( ) | ( ) | ( ) |

**During the pandemic, which employee groups were offered the option of remote work, commonly referred to as “home office”?**

( ) Non-clinical staff only

( ) Clinical staff only

( ) Both clinical and non-clinical staff

( ) No staff

**Based on the experience gained during the COVID-19 pandemic, is the healthcare provider planning to implement changes in staff care?**

( ) Yes

( ) No

( ) I don't know, I'm not sure

**These staff care changes will be:**

( ) General (applicable to all employees)

( ) Specific (applicable to selected groups of employees only)

( ) Both general (some applicable to all employees) and specific (some applicable only to selected employee groups only)

( ) I don't know, I'm not sure

**(Optional) Please describe what changes in staff care are planned:**

..........................................................................................................................

..........................................................................................................................

**Based on the experience gained during the COVID-19 pandemic, is the healthcare provider planning to implement changes in the area of mental health services for staff?**

( ) Yes

( ) No

( ) I don't know, I'm not sure


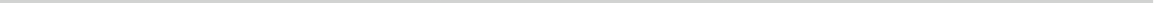


# Page 5 - Infrastructure and Material preparedness

**Have there been any infrastructure modifications in hospital departments during the pandemic (construction of isolation rooms, installation of specialized equipment - HEPA filters, airlock doors, etc.)?**

( ) Yes

( ) No

( ) I'm not sure

**Have there been any infrastructure modifications on the hospital premises during the pandemic (modification of entrances and changes in other common areas, etc.)?**

( ) Yes

( ) No

( ) I don't know, I'm not sure

**Will or were these infrastructural changes kept in place after the end of the COVID19 pandemic?**

( ) Yes, all of them,

( ) Yes, some / partially

( ) No, none

( ) I don't know, I'm not sure

**(Optional) Please describe what infrastructural changes were made:**

..........................................................................................................................

..........................................................................................................................

**Based on the experience gained during the COVID-19 pandemic, is the health care provider planning to incorporate requirements for multi-purpose buildings into its development plans? (multi-purpose buildings are designed for multiple uses, such as a standard ward equipped with reinforced oxygen supply that can be converted to infection unit)**

( ) Yes

( ) No

( ) I don't know, I'm not sure

**Based on the experience gained during the COVID-19 pandemic, have changes to the development plans (changes to building plans,...) been or will be made?**

( ) Yes

( ) No

( ) I don't know, I'm not sure

**(Optional) Please describe what changes to the development plans have been or are to be made:**

..........................................................................................................................

..........................................................................................................................

**Based on the experience gained during the COVID-19 pandemic, is the healthcare provider planning to implement changes in patient admission processes?**

( ) Yes

( ) No

( ) I don't know, I'm not sure

**(Optional) Please describe what changes in patient admission processes are planned:**

..........................................................................................................................

..........................................................................................................................

**Based on the experience gained during the COVID-19 pandemic, is the healthcare provider planning to implement changes in bed management system?**

( ) Yes

( ) No

( ) I don't know, I'm not sure

**(Optional) Please describe what changes in bed management system are planned:**

.......................................................................................................................... ..........................................................................................................................

**Has the healthcare provider implemented a real-time bed occupancy monitoring system?**

( ) Yes

( ) No

( ) I don't know, I'm not sure

**Has the healthcare provider implemented a standardized protocol for transferring patients to another facility or healthcare provider in cases of insufficient bed capacity?**

( ) Yes

( ) No

( ) I don't know, I'm not sure

**Based on the experience gained during the COVID-19 pandemic, is the healthcare provider planning to implement changes in medical supplies (respirators, gowns, etc.) management?**

( ) Yes

( ) No

( ) I don't know, I'm not sure

**(Optional) Please describe what changes in medical supplies (respirators, gowns, etc.) management are planned:**

..........................................................................................................................

..........................................................................................................................

**Does the healthcare provider communicate the stock status of medical supplies (respirators, gowns, etc.) with:**

|  | Yes | No |
| --- | --- | --- |
| Employees | ( ) | ( ) |
| Other healthcare providers | ( ) | ( ) |
| Governmental institutions | ( ) | ( ) |

**Has the healthcare provider implemented a standardized protocol for the exchange of medical supplies with another healthcare provider?**

( ) Yes

( ) No

( ) I don't know, I'm not sure

**Based on the experience gained during the COVID-19 pandemic, is the healthcare provider planning to implement changes in the medical equipment management (ventilators, dialysis machines, etc.)?**

( ) Yes

( ) No

( ) I don't know, I'm not sure

**(Optional) What changes in medical equipment (ventilators, dialysis machines, etc.) management are planned?**

..........................................................................................................................

..........................................................................................................................

**Does the healthcare provider communicate about the stock status of medical equipment (ventilators, dialysis machines, etc.) with:**

|  | Yes | No |
| --- | --- | --- |
| Employees | ( ) | ( ) |
| Other healthcare providers | ( ) | ( ) |
| Governmental institutions | ( ) | ( ) |

**Has the healthcare provider implemented a standardized protocol for the exchange of medical equipment (ventilators, dialysis machines, etc. ) with another healthcare provider?**

( ) Yes

( ) No

**Has the healthcare provider implemented a standardized protocol to secure necessary medical supplies and medical equipment in case of acute shortages during crisis situations?**

( ) Yes

( ) No


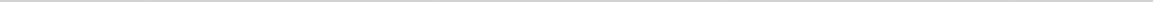


# Page 6 - Other impacts of the pandemic

**Based on the experience gained during the COVID-19 pandemic, is the healthcare provider planning to make changes in any of the following areas:**

|  | Yes | No | I don't know, I'm not sure |
| --- | --- | --- | --- |
| Infection prevention and control system | ( ) | ( ) | ( ) |
| Management or structure of medical records | ( ) | ( ) | ( ) |
| Placement of medical equipment in the wards | ( ) | ( ) | ( ) |
| Staff training | ( ) | ( ) | ( ) |
| Internal communication strategy | ( ) | ( ) | ( ) |
| Electronization of processes or use of telemedicine applications | ( ) | ( ) | ( ) |
| Operational data collection and reporting | ( ) | ( ) | ( ) |
| Communication strategy towards other healthcare providers or Emergency Medical Services providers | ( ) | ( ) | ( ) |
| Public communication strategy | ( ) | ( ) | ( ) |
| Collaboration with scientific or private institutions | ( ) | ( ) | ( ) |

**Additional comments on the areas:**

1. Infection prevention and control system
2. Management or structure of medical records
3. Placement of medical equipment in the wards
4. Staff training
5. Internal communication strategy
6. Electronization of processes or use of telemedicine applications
7. Operational data collection and reporting
8. Communication strategy towards other healthcare providers or Emergency Medical Services providers
9. Public communication strategy
10. Collaboration with scientific or private institutions

..........................................................................................................................

..........................................................................................................................


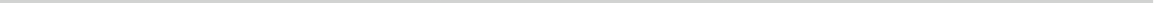


# Page 7 - Thank you and farewell

**Thank you for your participation in this survey. If you have any questions or feedback, please don't hesitate to contact us. We appreciate your contribution to our research.**

**On behalf of the research team**

**--**

**XXX**

**XXX**

**XXX**


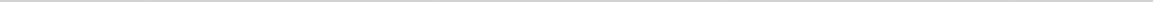

Supplement: Supplementary file 1 [file DataSheet2.docx]
