# Supplementary material for: Hospital-Level COVID-19 Preparedness and Crisis Management in Czechia
Source: Int J Public Health. 2023 Dec 14;68:1606398. doi: 10.3389/ijph.2023.1606398 (PMC10752954; doi:10.3389/ijph.2023.1606398)
Supplement: Supplementary file 2 [file DataSheet1.docx]

**Supplementary file 1**

***Supplementary file 1:*** *Search parameters for the semi-systematic review on crisis preparedness literature used in the article preparations*

Parameters:

A) Time period: 2019-2022

B) Limiters: ( "crisis response" OR "crisis resilience" OR "crisis resources management" OR "crisis preparedness" OR "healthcare resilience" OR "crisis planning" OR "emergency planning" OR "crisis capacity" OR "hazard analysis" OR "hazard vulnerability analysis" OR "hazard mitigation" OR "capability assessment" OR "hazard assessment" OR "disaster planning") AND ( covid-19 or coronavirus or 2019-ncov or sars-cov-2 or cov-19) AND(“hospital”), Full text

C) Language: English

D) Platforms: EBSCOhost (all databases), PUBMED

Results:

- EBSCOhost (all databases) – 131 results
- PUBMED – 275 results

Further excluded:

- Retracted articles – 2
- Duplicate article – 114
- Non-relevant (after the title assesment) – 48

Total analysed: 242 articles
